# Interventions for Persistent Pain Despite Low Inflammatory Activity in Rheumatoid Arthritis: A Systematic Review

**DOI:** 10.7759/cureus.102091

**Published:** 2026-01-22

**Authors:** Ryuichi Ohta, Taichi Fujimori, Kunihiro Ichinose

**Affiliations:** 1 Community Care, Unnan City Hospital, Unnan, JPN; 2 Rheumatology, Shimane University Faculty of Medicine, Izumo, JPN

**Keywords:** chronic pain, disease activity, disease-modifying antirheumatic drugs, exercise therapy, omega-3 fatty acids, quality of life, rheumatoid arthritis

## Abstract

Persistent pain remains a significant clinical challenge in patients with rheumatoid arthritis (RA), even after achieving normal or low inflammatory activity. This discordance between inflammation control and pain relief suggests the involvement of mechanisms beyond active synovitis, yet the effectiveness of available interventions in this population has not been fully clarified. We conducted a systematic review to synthesize evidence on pharmacological and non-pharmacological interventions for persistent pain in RA patients with controlled inflammatory activity. Major biomedical databases were searched for studies reporting pain-related outcomes in adult RA patients, including randomized controlled trials, post hoc analyses of clinical trials, and prospective observational studies. Owing to heterogeneity in study design, interventions, and outcome measures, a narrative synthesis was performed. Eight studies met the inclusion criteria. Pharmacological interventions targeting inflammatory pathways, including conventional synthetic disease-modifying antirheumatic drugs (DMARDs), biologic agents such as tumor necrosis factor and interleukin-6 receptor inhibitors, and targeted synthetic DMARDs such as Janus kinase inhibitors, demonstrated heterogeneous and generally limited effects on pain reduction once inflammation was controlled, with clinically meaningful pain frequently persisting despite improvements in disease activity. In contrast, non-pharmacological interventions, particularly exercise-based and physical therapy-oriented approaches such as nerve mobilization and structured physical activity programs, showed more consistent reductions in pain intensity independent of inflammatory markers and were often accompanied by functional improvement. Observational evidence also suggested that lifestyle-related factors, including dietary intake of omega-3 fatty acids, may be associated with pain outcomes independently of inflammatory control. Overall, these findings indicate that persistent pain in RA patients with normal or low inflammatory activity is only partially responsive to inflammation-targeted pharmacological therapy, supporting the integration of pharmacological treatment with exercise-based and lifestyle-oriented strategies within comprehensive, mechanism-informed pain management approaches.

## Introduction and background

Rheumatoid arthritis (RA) is a chronic inflammatory disease characterized by progressive joint destruction, functional impairment, and reduced quality of life (QOL). The primary goal of RA management has traditionally focused on controlling inflammation to prevent structural damage and disability [1]. With the development and widespread use of disease-modifying antirheumatic drugs (DMARDs), including biologic agents and Janus kinase inhibitors, an increasing proportion of patients now achieve low disease activity or clinical remission as defined by composite indices and inflammatory markers [2]. As a result, modern RA care has successfully reduced radiographic progression and improved long-term functional outcomes in many patients.

Despite these therapeutic advances, a substantial subset of patients continues to experience moderate to severe pain even when inflammatory activity is well controlled. Clinical observations and epidemiological studies have shown that pain, fatigue, and functional limitations persist in a considerable proportion of patients who meet criteria for remission or low disease activity, including those with normal or near-normal C-reactive protein (CRP) levels [3-5]. This phenomenon, often described as “residual pain” or “discordant pain,” highlights a clinically meaningful mismatch between objective inflammatory measures and patient-reported pain outcomes [5,6]. Such discordance poses a challenge to treat-to-target strategies that primarily emphasize inflammation suppression and disease activity scores.

The persistence of pain under low inflammatory conditions suggests that RA-related pain is not solely driven by peripheral inflammation. Several mechanisms have been proposed to explain this discrepancy, including concomitant fibromyalgia, central sensitization with amplified pain processing, irreversible structural joint damage, and psychological or psychosocial factors, such as depression, anxiety, and pain catastrophizing [6]. These factors may contribute independently or synergistically to sustained pain, even when synovitis is adequately controlled. Consequently, continued escalation or switching of anti-inflammatory therapies in these patients does not always lead to meaningful pain relief and may expose patients to unnecessary treatment-related risks without addressing the underlying pain mechanisms.

A wide range of pharmacological and non-pharmacological interventions has been explored to manage pain in RA, including analgesic adjuvants such as serotonin-norepinephrine reuptake inhibitors and gabapentinoids, exercise therapy, cognitive behavioral therapy, and multidisciplinary chronic pain management programs [7]. However, evidence regarding the effectiveness of these interventions, specifically in RA patients with low inflammatory activity or normal CRP levels, remains fragmented. Many studies have examined heterogeneous RA populations, reported pain outcomes as secondary endpoints, or conducted subgroup analyses without focusing explicitly on patients with discordant pain. Moreover, variations in pain definitions, inflammatory thresholds, and outcome measures across studies further complicate the interpretation and clinical application of existing evidence [8].

Given these gaps, a comprehensive synthesis of the available evidence focusing on RA patients with persistent pain despite well-controlled inflammation is needed. The present systematic review aimed to evaluate the effectiveness of pharmacological and non-pharmacological interventions for pain management in RA patients with normal or low inflammatory activity, with particular attention to pain, functional outcomes, and QOL. By systematically organizing current evidence, this review seeks to clarify therapeutic options for this clinically challenging population and to provide a foundation for individualized, mechanism-informed pain management strategies in RA.

## Review

Study design

This study was conducted as a systematic review to synthesize existing evidence on the effectiveness of interventions for persistent pain in patients with RA who exhibit normal or low inflammatory activity. The review was designed and conducted in accordance with the Preferred Reporting Items for Systematic Reviews and Meta-Analyses (PRISMA) guidelines [9]. Given the anticipated heterogeneity in study designs, intervention types, pain definitions, and outcome measures, a narrative synthesis was planned as the primary method of data integration, with quantitative synthesis considered where appropriate. Before initiating the review process, the study protocol was prospectively registered with the International Prospective Register of Systematic Reviews (PROSPERO; registration ID: CRD420251244917).

Data sources and search strategy

A comprehensive literature search was performed using major biomedical databases, including PubMed, Embase, and Web of Science. These databases were selected to ensure broad coverage of clinical, pharmacological, and rehabilitation-related studies relevant to RA and pain management.

The search strategy combined controlled vocabulary terms (e.g., MeSH and Emtree terms) and free-text keywords related to rheumatoid arthritis, inflammatory activity, pain, and therapeutic interventions. Core search concepts included rheumatoid arthritis, C-reactive protein, low disease activity, remission, pain, residual pain, discordant pain, and treatment. Keywords related to pharmacological interventions (e.g., DMARDs, analgesic adjuvants) and non-pharmacological therapies (e.g., exercise therapy, cognitive behavioral therapy, multidisciplinary care) were incorporated as appropriate.

Electronic Search Strategy

A comprehensive literature search was conducted in PubMed, Embase, and Web of Science to identify studies evaluating interventions for persistent pain in patients with rheumatoid arthritis with normal or low inflammatory activity. The PubMed search strategy was as follows: ("rheumatoid arthritis"[MeSH Terms] OR "rheumatoid arthritis"[Title/Abstract] OR RA[Title/Abstract]) AND ("pain"[MeSH Terms] OR pain[Title/Abstract] OR "persistent pain"[Title/Abstract] OR "residual pain"[Title/Abstract] OR "discordant pain"[Title/Abstract]) AND ("low disease activity"[Title/Abstract] OR remission[Title/Abstract] OR "low inflammatory activity"[Title/Abstract] OR "normal CRP"[Title/Abstract] OR "C-reactive protein"[MeSH Terms]) AND (exercise[Title/Abstract] OR "physical therapy"[Title/Abstract] OR "nerve mobilization"[Title/Abstract] OR DMARD[Title/Abstract] OR biologic[Title/Abstract] OR "Janus kinase inhibitor*"[Title/Abstract])**

Equivalent search strategies were adapted for Embase using Emtree terms and for Web of Science using keyword-based searches. The searches were conducted without restrictions on publication date, and studies published in English were eligible for inclusion. Reference lists of relevant reviews and included studies were manually screened to identify additional eligible articles not captured by the electronic database searches.

The search period was not restricted by publication date; however, studies published from the year 2000 onward were prioritized to reflect contemporary RA management strategies. Only articles published in English were included. Reference lists of relevant reviews and eligible studies were manually screened to identify additional publications not captured by the database search. Grey literature, including conference abstracts and unpublished studies, was not routinely included due to concerns regarding data completeness and methodological rigor.

Eligibility criteria

Eligibility criteria were predefined based on the Population, Intervention, Comparator, and Outcome (PICO) framework. Studies were included if they involved adult patients diagnosed with rheumatoid arthritis and explicitly addressed persistent pain despite low inflammatory disease activity, as defined by clinical assessment or composite disease activity indices. Both qualitative and quantitative studies were considered, including observational studies, interventional trials, and mixed-methods research. Studies focusing exclusively on high inflammatory activity, pediatric populations, or non-rheumatoid inflammatory arthritis were excluded.

Inclusion Criteria

Studies were eligible for inclusion if they examined adult patients aged 18 years or older with RA. To ensure consistency with the focus of this review, eligible studies were required to include patients with normal or low levels of inflammatory activity. Low inflammatory status was defined as normal or low CRP levels, or as achieving low disease activity or remission according to validated disease activity indices.

In addition to inflammatory status, studies were required to explicitly report the presence of persistent pain of at least moderate severity, operationalized as a visual analog scale (VAS) or numerical rating scale (NRS) pain score ≥30 mm (or ≥3/10), or to describe a clinically meaningful discordance between pain and inflammatory activity (e.g., residual or pain-inflammation discordance), as defined by the original study authors. Studies using alternative, validated pain thresholds or categorical definitions (e.g., unacceptable pain) were included, provided that pain remained clearly present despite normal or low levels of inflammatory activity.

Studies were included if they investigated interventions targeting this clinical condition, encompassing both pharmacological and non-pharmacological approaches. Pharmacological interventions included optimization or modification of DMARDs as well as agents intended to modulate pain perception. In contrast, non-pharmacological interventions included exercise therapy, cognitive behavioral therapy, and multidisciplinary pain management programs.

Eligible studies were required to report pain-related outcomes assessed using validated measurement tools. Studies reporting additional outcomes related to physical function or quality of life were also included. A broad range of study designs was considered acceptable, including randomized controlled trials, non-randomized interventional studies, observational studies, and before-and-after studies, provided that sufficient methodological detail was available to allow appropriate interpretation of the findings.

Exclusion Criteria

Studies were excluded if they were case reports, narrative reviews, systematic reviews, editorials, or expert opinion articles. Publications that did not report pain-related outcomes or that lacked sufficient information regarding inflammatory activity were also excluded. In addition, studies were excluded if the study population did not primarily consist of patients with rheumatoid arthritis.

Studies focusing exclusively on patients with high inflammatory activity were excluded unless subgroup analyses specifically addressing patients with low or normal inflammatory status were available. Conference abstracts and unpublished reports without accessible full-text articles were excluded due to insufficient methodological detail and outcome reporting. Furthermore, studies published in languages other than English were not considered eligible for inclusion.

Study selection

All identified records were imported into a reference management system, and duplicate entries were removed. Two reviewers independently screened titles and abstracts to assess eligibility according to the predefined criteria. Full-text articles were retrieved for studies deemed potentially eligible. Discrepancies between reviewers at any stage were resolved through discussion, and when consensus could not be reached, a third reviewer was consulted to reach a final decision.

Data extraction

Data extraction was conducted using a standardized extraction form developed prior to the review. Two reviewers independently extracted data from each included study, with discrepancies resolved by consensus. Extracted data included study characteristics (author, year, country, and study design), patient demographics, disease duration, inflammatory markers, pain definitions and measurement tools, intervention details (type, dosage, duration), comparator information where applicable, outcome measures related to pain, function, and QOL, follow-up duration, and reported adverse events.

Information regarding comorbid conditions, such as fibromyalgia, indicators of central sensitization, and psychological factors, was extracted when available. When data were incomplete or unclear, the study was included in qualitative synthesis if sufficient contextual information was provided.

Data synthesis

Given the expected heterogeneity across studies in patient populations, intervention modalities, and outcome measures, a narrative synthesis was used as the primary method of data integration. Studies were grouped by intervention type as follows: inflammatory treatment optimization, pharmacological pain-modulating therapies, and non-pharmacological interventions.

Where multiple studies with comparable designs and outcome measures were identified, the feasibility of quantitative synthesis using random-effects models was assessed. Heterogeneity was evaluated using the I^2^ statistic. When meta-analysis was not feasible, results were summarized descriptively, with emphasis on clinical relevance and consistency of findings across studies.

Study management and risk of bias assessment

Study screening, selection, and data management were conducted using Covidence systematic review software (Melbourne, Australia: Veritas Health Innovation) [10]. Risk of bias in randomized controlled trials was assessed using the Cochrane Risk of Bias tool version 2 (RoB 2), while the methodological quality of observational studies was evaluated using the Newcastle-Ottawa Scale (NOS) [11,12]. This systematic review was conducted and reported in accordance with the PRISMA 2020 guidelines.

Results

Study Selection

**Figure 1:**
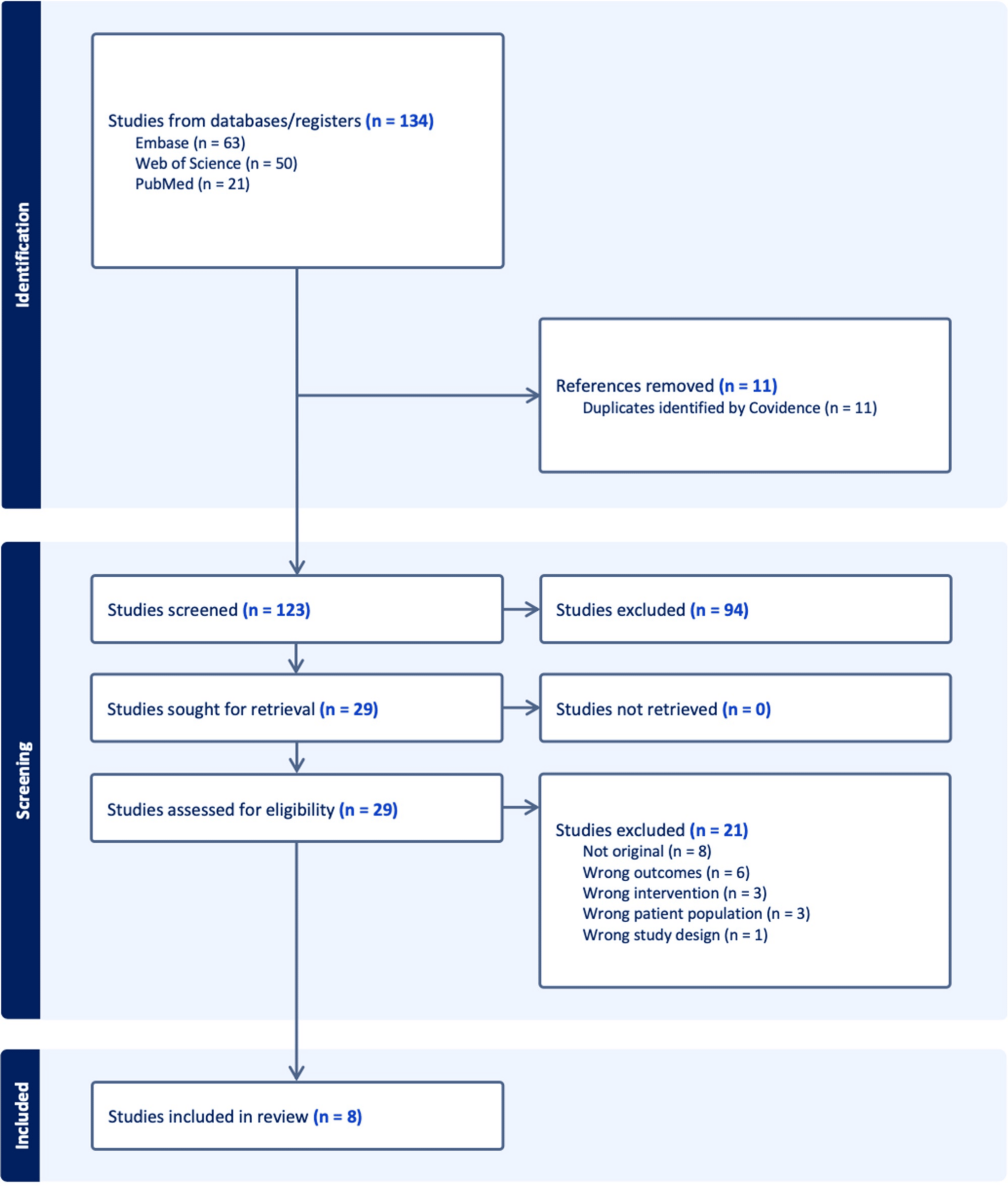
PRISMA flow diagram illustrating the study selection process for the systematic review. PRISMA: Preferred Reporting Items for Systematic Reviews and Meta-Analyses

All records identified through database searches were imported into Covidence systematic review software for study management and screening. A total of 134 records were identified from electronic databases, including Embase (n=63), Web of Science (n=50), and PubMed (n=21). After removal of duplicate records identified by Covidence (n=11), 123 unique records remained for title and abstract screening. During the initial screening phase, titles and abstracts were independently reviewed by two reviewers to assess potential eligibility. Of the 123 records screened, 94 were excluded based on predefined eligibility criteria. The most common reasons for exclusion at this stage included non-original articles (n=8), studies reporting outcomes unrelated to pain (n=6), interventions not relevant to the research question (n=3), studies involving an inappropriate patient population (n=3), and unsuitable study designs (n=1). Additional records were excluded because they did not meet the inclusion criteria. Following title and abstract screening, 29 articles were deemed potentially eligible and were retrieved in full for detailed evaluation. All 29 full-text articles were successfully retrieved and assessed for eligibility. After full-text review, 21 articles were excluded for reasons consistent with the exclusion criteria, including lack of relevant pain outcomes, inappropriate interventions, or failure to define inflammatory status clearly. Ultimately, eight studies met all inclusion criteria and were included in the final qualitative synthesis. No studies were excluded due to the inability to retrieve full-text articles. The study selection process is summarized in the PRISMA flow diagram (Figure 1).

Characteristics of the Included Articles

A total of eight studies met the predefined eligibility criteria and were included in the final qualitative synthesis. The included studies were published between 2017 and 2025, reflecting recent advances in RA management. Study designs were heterogeneous and included post hoc analyses of randomized controlled trials (n=3), randomized controlled pilot trials (n=1), prospective interventional studies (n=1), and prospective observational cohort or registry-based studies (n=3). This diversity in study design reflects the limited availability of randomized trials specifically targeting persistent pain in RA patients with low inflammatory activity.

The study populations consisted exclusively of adult patients with established RA. Sample sizes varied widely, ranging from a small, randomized pilot study with 12 participants to large post hoc or registry-based analyses including more than 3,000 patients. The mean or median age of participants across studies generally ranged from the early 50s to early 60s, and all studies reported a predominance of female patients, consistent with the epidemiology of RA. Across all included studies, patients were characterized by normal or low inflammatory activity, most commonly defined by normal or low CRP levels, Disease Activity Score-28 (DAS28) indicating low disease activity, or clinical remission.

Despite adequate inflammatory control, all included studies explicitly reported persistent pain of at least moderate severity or described a discordance between pain intensity and inflammatory markers. Several studies operationalized this phenotype using predefined thresholds of pain severity. In contrast, others described it as residual pain or as pain-inflammation discordance, indicating that pain remained clinically relevant despite suppression of inflammatory activity.

Pain outcomes were assessed using validated patient-reported measures, including visual analog scales (VAS), numerical rating scales (NRS), and pain components embedded within composite disease activity indices. In addition to pain, several studies reported secondary outcomes related to physical function or disability, most commonly using validated functional indices, and quality of life, assessed with standardized instruments. However, the specific outcome measures used and the timing of assessments varied substantially across studies, contributing to methodological heterogeneity.

The interventions evaluated encompassed both pharmacological and non-pharmacological approaches. Pharmacological interventions investigated in post hoc analyses and cohort studies included optimization or modification of disease-modifying antirheumatic drugs, such as methotrexate, biologic DMARDs, and targeted synthetic DMARDs, as well as comparisons of different therapeutic strategies. Non-pharmacological interventions, evaluated in interventional studies, included exercise-based programs, nerve mobilization techniques, and structured physical activity interventions. The duration of interventions and follow-up periods ranged from short-term assessments lasting several weeks or months to longer follow-ups extending up to two years.

Information regarding comorbid conditions and pain-related modifiers was inconsistently reported across studies. Some studies evaluated the presence of fibromyalgia, features suggestive of central sensitization, or psychological factors, such as anxiety or depression, whereas others did not systematically assess these variables. This variability limited direct comparison across studies but underscored the multifactorial nature of persistent pain in RA patients with well-controlled inflammatory activity (Table 1).

**Table 1:** Demographic data of the included articles. Characteristics of the eight studies included in this systematic review evaluating pharmacological and non-pharmacological interventions for persistent pain in patients with rheumatoid arthritis and normal or low inflammatory activity. The table summarizes the study authors, publication year, country, study design, sample size, intervention type, and duration of follow-up. Included studies comprise randomized controlled pilot studies, post hoc analyses of phase 3 randomized controlled trials, prospective cohort studies, and nationwide registry-based observational studies. Interventions ranged from non-pharmacological approaches, such as nerve mobilization, structured physical activity, and dietary assessment, to pharmacological therapies including conventional synthetic, biologic, and targeted synthetic disease-modifying antirheumatic drugs. Follow-up duration varied from short-term assessments over several weeks to long-term observations extending up to two years. RA: rheumatoid arthritis; RCT: randomized controlled trial; MTX: methotrexate; bDMARD: biologic disease-modifying antirheumatic drug; csDMARD: conventional synthetic disease-modifying antirheumatic drug; tsDMARD: targeted synthetic disease-modifying antirheumatic drug; JAK: Janus kinase; FFQ: food frequency questionnaire; TNF: tumor necrosis factor

Studies	Year	Country	Design	Sample size	Intervention	Duration	Outcome
Lo et al. [13]	2017	Singapore/Hong Kong	Randomized controlled pilot study (nerve mobilization vs. joint mobilization)	12	Nerve mobilization	4 weeks	Nerve mobilization resulted in greater pain reduction than joint mobilization despite no change in inflammatory markers
Löfgren et al. [14]	2018	Sweden	Prospective 2-year physical activity intervention follow-up	30	Strength training + moderate aerobic activity + behavioral support	2 years	Long-term physical activity was associated with reduced pain intensity, independent of disease activity
Lourdudoss et al. [15]	2018	Sweden	Population-based prospective cohort with MTX monotherapy follow-up	591	Dietary intake (omega-3, omega-6; FFQ-based) during early RA, observational assessment	3 months follow-up after MTX initiation	Higher omega-3 intake was associated with lower pain despite inflammatory control under MTX therapy
Fautrel et al. [16]	2019	Multinational (France, UK, Canada, Japan, USA)	Post hoc analysis of phase 3 RCT	1,010	Baricitinib 4 mg daily + MTX adalimumab 40 mg every 2 weeks + MTX Placebo + MTX	24 weeks (primary timepoint for this analysis)	Substantial residual pain persisted in patients with low disease activity across all treatment arms
Olofsson et al. [17]	2021	Sweden	Prospective longitudinal cohort comparing bDMARD vs. csDMARD initiators	1,307	Initiation of bDMARD or csDMARD therapy in routine care	24 months	Persistent pain remained common despite low inflammatory activity, regardless of bDMARD or csDMARD initiation
Dougados et al. [18]	2022	Multinational	Post hoc analysis of 9 RCTs	3,588	Tofacitinib 5 mg twice daily	3 months	Pain improved with tofacitinib, but pain-inflammation discordance persisted in a subset of patients
Kim et al. [19]	2022	Korea	Nationwide prospective registry	2,379	Biologics (anti-TNF, tocilizumab, abatacept, rituximab) or JAK inhibitors (tsDMARDs); no randomized assignment	Baseline to 1-year registry follow-up	Clinically relevant pain frequently persisted despite remission or low disease activity in real-world practice
Tanaka et al. [20]	2024	Japan	Post hoc analysis of a phase 3 RCT	91	Sarilumab monotherapy (150 mg or 200 mg q2w), sarilumab + non-MTX csDMARDs (same dosing), dose: 150 mg or 200 mg every 2 weeks, duration: 52 weeks	52 weeks	Sarilumab reduced unacceptable pain in some patients with low inflammatory activity, with variable individual response

Effects of pharmacological interventions

Biologic and Targeted Synthetic DMARDs

Several included studies evaluated the effects of biologic and targeted synthetic DMARDs on pain outcomes in patients with rheumatoid arthritis who had achieved normal or low inflammatory activity. These studies primarily consisted of post hoc analyses of randomized controlled trials and large prospective observational cohorts, allowing assessment of pain outcomes under well-controlled inflammation [16,18,19].

In the post hoc analysis of one randomized controlled trial, Fautrel et al. examined pain outcomes in patients treated with baricitinib plus methotrexate, adalimumab plus methotrexate, or placebo plus methotrexate. Among patients who achieved low disease activity, pain improvement was observed across treatment groups; however, a substantial proportion of patients continued to report clinically relevant pain despite adequate suppression of inflammatory activity [16]. Differences in pain reduction between treatment arms were modest, suggesting that further escalation of anti-inflammatory therapy yielded limited additional benefit for pain once disease activity was controlled.

Targeted synthetic DMARDs were further evaluated in a large post hoc analysis of nine randomized controlled trials by Dougados et al., focusing on patients treated with tofacitinib [18]. Although tofacitinib treatment was associated with improvements in pain scores at three months, pain reduction was not consistently aligned with changes in disease activity measures. A subset of patients continued to experience disproportionate pain despite low inflammatory activity, indicating heterogeneity in pain response to JAK inhibition.

Real-world evidence from a nationwide prospective registry was provided by Kim et al., who analyzed pain outcomes in patients initiating biologic DMARDs or targeted synthetic DMARDs, including anti-tumor necrosis factor (TNF) agents, tocilizumab, abatacept, rituximab, and JAK inhibitors [19]. Over one year of follow-up, improvements in disease activity were observed across treatment groups; however, persistent pain remained common among patients who achieved low inflammatory activity. No single biologic or targeted synthetic DMARD class consistently eliminated pain in this population, highlighting variability in individual pain responses.

Interleukin-6 receptor inhibition was specifically examined in a post hoc analysis of one phase 3 randomized controlled trial by Tanaka et al. [20]. In this study, patients receiving sarilumab monotherapy or sarilumab combined with non-methotrexate conventional synthetic DMARDs demonstrated reductions in pain scores over 52 weeks. Importantly, pain improvement was observed even among patients with low inflammatory activity, suggesting a potential effect of IL-6 blockade on pain mechanisms beyond peripheral inflammation. Nevertheless, the magnitude of pain reduction varied across individuals and did not uniformly translate into parallel improvements in functional outcomes.

Conventional Synthetic DMARD Optimization

The effects of conventional synthetic DMARD therapy on pain outcomes were primarily assessed in observational cohort studies. In a large population-based prospective cohort study from Sweden, Olofsson et al. compared patients initiating biologic DMARDs with those initiating conventional synthetic DMARDs in routine clinical care [17]. Although improvements in disease activity were observed in both groups over 24 months, persistent pain remained prevalent, particularly among patients who achieved low inflammatory activity. Initiation or optimization of conventional synthetic DMARD therapy alone was not sufficient to resolve pain in a substantial proportion of patients.

Similarly, Lourdudoss et al. examined early rheumatoid arthritis patients receiving methotrexate monotherapy and evaluated the association between dietary intake and pain outcomes [15]. Higher intake of omega-3 fatty acids was associated with lower pain levels during follow-up, whereas omega-6 intake showed no consistent association with pain outcomes. Importantly, no dietary intervention was performed, and dietary intake was assessed observationally using a food frequency questionnaire. Despite standardized methotrexate treatment and improvements in inflammatory markers, pain outcomes showed considerable variability, and many patients continued to report pain during follow-up. These findings suggest that dietary factors may modulate pain independently of inflammatory control, and they also support the limited capacity of conventional DMARD therapy alone to fully address persistent pain once inflammation is adequately controlled.

Effects of non-pharmacological interventions

Exercise-Based Interventions

Exercise-based interventions were evaluated in patients with rheumatoid arthritis who experienced persistent pain despite normal or low inflammatory activity in two included studies [13,14]. These interventions encompassed structured physical activity programs implemented over short- to long-term periods.

In a randomized controlled pilot study, Lo et al. compared nerve mobilization with joint mobilization over a four-week intervention period [13]. Patients receiving nerve mobilization demonstrated greater reductions in pain intensity compared with those receiving joint mobilization, despite no significant changes in inflammatory markers. This finding suggests that targeted physical interventions addressing neural mechanosensitivity may reduce pain independently of inflammation control.

Löfgren et al. investigated a two-year physical activity intervention combining strength training, moderate aerobic exercise, and behavioral support in a subsample of the RCT study [14]. Over the long-term follow-up period, participants showed reductions in pain intensity alongside improvements in physical function. Notably, these improvements occurred without corresponding changes in inflammatory disease activity, indicating that sustained physical activity may alleviate pain through non-inflammatory mechanisms.

Across these studies, exercise-based interventions were associated with reductions in pain intensity, as measured with validated patient-reported outcome measures. The magnitude of pain improvement varied between studies, likely reflecting differences in intervention content, intensity, duration, and baseline patient characteristics. Nevertheless, no serious adverse events related to exercise interventions were reported, supporting their safety and tolerability in patients with controlled inflammatory disease.

Cognitive and Behavioral Interventions

Explicit cognitive-behavioral therapy-based interventions were not evaluated independently in the included studies. However, behavioral components were incorporated into the long-term physical activity intervention examined by Löfgren et al. [14]. In this study, behavioral support aimed at promoting physical activity adherence was associated with sustained improvements in pain outcomes over two years.

The observed pain reduction occurred in the absence of measurable changes in inflammatory markers, supporting the contribution of central and psychosocial mechanisms to pain persistence in rheumatoid arthritis. Although psychological outcomes were not systematically assessed across studies, the findings suggest that behavioral strategies integrated within physical activity programs may enhance pain coping and contribute to long-term pain improvement.

Multidisciplinary and Physical Therapy-Based Approaches

Multidisciplinary or physical therapy-based approaches were represented by interventions combining physical techniques with patient education or behavioral elements. Lo et al. demonstrated that nerve mobilization, a targeted physical therapy technique, produced greater pain reduction than joint mobilization alone, highlighting the potential value of mechanism-oriented physical therapy strategies [13].

Similarly, the intervention evaluated by Löfgren et al. integrated exercise training with behavioral support, reflecting a multidimensional approach to pain management [14]. This combined strategy was associated with improvements not only in pain but also in functional capacity over extended follow-up.

Across these studies, improvements in pain were often accompanied by functional benefits, whereas quality of life outcomes were less consistently reported. Adverse events related to non-pharmacological interventions were infrequent and mild, reinforcing the tolerability of these approaches in patients with rheumatoid arthritis and controlled inflammatory activity.

Quality assessment

Risk of Bias Assessment

The results of the risk of bias assessment are summarized in Table 2. Overall, randomized controlled studies demonstrated a low to moderate risk of bias, with limitations primarily related to lack of blinding due to the nature of physical interventions. Observational and registry-based studies showed moderate methodological quality, with strengths including large sample sizes and prospective data collection, but with potential risks related to confounding and selection bias.

**Table 2:** Risk of bias assessment of included studies. Table 2 summarizes the risk of bias assessment of the studies included in this systematic review. Randomized controlled trials and randomized pilot studies were evaluated using the Cochrane Risk of Bias tool version 2 (RoB 2), assessing potential bias related to selection, performance, detection, attrition, and reporting domains. Observational cohort and registry-based studies were assessed using the Newcastle-Ottawa Scale (NOS), which evaluates methodological quality based on participant selection, comparability of study groups, and outcome assessment. RCT: randomized controlled trial; RoB: risk of bias; RoB 2: Cochrane Risk of Bias tool version 2; NOS: Newcastle-Ottawa Scale

Studies	Year	Study design	Risk of bias tool	Selection bias	Performance bias	Detection bias	Attrition bias	Reporting bias	Overall risk of bias
Lo et al. [13]	2017	Randomized controlled pilot study	Cochrane RoB 2	Low	Some concerns	Low	Low	Low	Moderate
Löfgren et al. [14]	2018	Prospective interventional study	Newcastle-Ottawa Scale	3	N/A	N/A	N/A	N/A	Moderate
Lourdudoss et al. [15]	2018	Prospective cohort study	Newcastle-Ottawa Scale	3	N/A	N/A	N/A	N/A	Moderate
Fautrel et al. [16]	2019	Post hoc analysis of RCT	Cochrane RoB 2	Low	Some concerns	Low	Low	Low	Moderate
Olofsson et al. [17]	2021	Prospective cohort study	Newcastle-Ottawa Scale	4	N/A	N/A	N/A	N/A	Low
Dougados et al. [18]	2022	Post hoc analysis of RCTs	Cochrane RoB 2	Low	Some concerns	Low	Low	Low	Moderate
Kim et al. [19]	2022	Nationwide registry-based study	Newcastle-Ottawa Scale	3	N/A	N/A	N/A	N/A	Moderate
Tanaka et al. [20]	2024	Post hoc analysis of phase 3 RCT	Cochrane RoB 2	Low	Some concerns	Low	Low	Low	Moderate

For randomized studies, the overall risk of bias was judged to be low to moderate. Random sequence generation and allocation concealment were generally well described in the original trial protocols; however, blinding of participants and personnel was limited or not feasible in studies involving physical interventions. Outcome assessment of pain was based on validated patient-reported outcome measures, which reduced detection bias, although reliance on self-reported outcomes may have introduced subjective variability.

Quality of Observational and Post Hoc Studies

Observational cohort studies and registry-based analyses demonstrated moderate methodological quality. Large sample sizes and prospective data collection strengthened internal validity in population-based and nationwide registry studies (e.g., Epidemiological Investigation of Rheumatoid Arthritis {EIRA} and Korean College of Rheumatology Biologics Registry {KOBIO} cohorts). However, the non-randomized nature of these studies introduced potential confounding, particularly confounding by indication and differences in baseline patient characteristics. Adjustment for disease activity and inflammatory markers was performed in several studies, but residual confounding could not be fully excluded.

Post hoc analyses of randomized controlled trials benefited from high-quality underlying trial data, including standardized outcome assessment and rigorous data collection. Nevertheless, because these analyses were not predefined primary endpoints, there was an inherent risk of selection and reporting bias. In addition, subgroup definitions of low inflammatory activity or pain-inflammation discordance varied across studies, which may have influenced comparability.

Heterogeneity and Outcome Measurement

Substantial heterogeneity was observed across studies in terms of study design, sample size, intervention type, duration of follow-up, and definitions of low inflammatory activity and persistent pain. Pain outcomes were measured using validated instruments, such as visual analog and numerical rating scales; however, thresholds for clinically meaningful pain and timing of outcome assessment differed across studies. This heterogeneity limited the feasibility of quantitative synthesis and justified the use of narrative synthesis as the primary method of data integration.

Discussion

Summary of the Study

In this systematic review, we synthesized evidence from eight studies examining interventions for persistent pain in patients with rheumatoid arthritis who had achieved normal or low inflammatory activity. Despite adequate control of inflammation, persistent pain remained common across diverse clinical settings and study designs. Pharmacological interventions targeting inflammatory pathways, including conventional, biologic, and targeted synthetic DMARDs, demonstrated limited and heterogeneous effects on pain reduction once inflammatory activity was controlled. In contrast, non-pharmacological interventions, particularly exercise-based and physical therapy-oriented approaches, showed more consistent improvements in pain outcomes, often without accompanying changes in inflammatory markers.

Notably, this systematic review also identified observational evidence suggesting that non-pharmacological factors, such as dietary intake, may be associated with pain outcomes independently of inflammatory control. Higher omega-3 fatty acid intake was associated with lower pain levels during methotrexate treatment in early rheumatoid arthritis. However, no dietary intervention was performed, and causal relationships could not be established. In addition, interleukin-6 receptor inhibition was associated with greater pain reduction than other pharmacological strategies in selected patient populations, although responses varied and pain relief was not universal. Overall, these findings highlight a substantial gap between inflammation control and pain resolution in rheumatoid arthritis and underscore the multifactorial nature of persistent pain in this population.

An important implication of these findings is the need to differentiate biological refractoriness from pain refractoriness in patients with rheumatoid arthritis. Biological refractoriness refers to persistent inflammatory activity despite appropriate disease-modifying antirheumatic therapy, whereas pain refractoriness describes ongoing pain that persists despite adequate suppression of inflammation. The present review suggests that a substantial proportion of patients with normal or low inflammatory activity fall into the latter category, in whom pain is not primarily driven by active synovitis. In such patients, persistent pain should not be automatically interpreted as evidence of uncontrolled disease activity. Misattributing pain refractoriness to residual inflammation may lead clinicians to escalate or switch immunosuppressive therapies unnecessarily, exposing patients to potential adverse effects while failing to address the dominant pain mechanisms.

Comparison With Other Studies

Our findings are consistent with previous reports describing discordance between inflammatory disease activity and patient-reported pain in rheumatoid arthritis [21]. Large cohort studies and post hoc trial analyses have repeatedly shown that a considerable proportion of patients continue to experience clinically meaningful pain despite achieving remission or low disease activity [22-24]. The present review extends these observations by focusing specifically on patients with normal or low inflammatory markers and by systematically comparing pharmacological and non-pharmacological interventions within this subgroup.

While pharmacological escalation has traditionally been the primary strategy for managing ongoing symptoms in rheumatoid arthritis, our results suggest that further suppression of inflammation alone may not adequately address pain once inflammatory control has been achieved [25]. The relatively greater pain reduction observed with IL-6 receptor inhibition aligns with emerging evidence that IL-6 may influence pain processing through central or peripheral mechanisms beyond synovial inflammation [26]. However, the variability in treatment response observed across studies emphasizes that no single pharmacological strategy uniformly resolves pain in this context.

In contrast, the beneficial effects of exercise-based and physical therapy-oriented interventions observed in this review are consistent with growing evidence supporting the role of non-pharmacological approaches in chronic pain management [27,28]. These findings parallel observations in other chronic inflammatory and non-inflammatory pain conditions, where physical activity and behavioral strategies improve pain and function independently of disease-specific biological markers. In addition, observational evidence suggests that lifestyle-related factors, such as dietary intake, may also be associated with pain outcomes independent of inflammatory activity, further supporting the concept that a broad range of non-inflammatory mechanisms influences persistent pain in rheumatoid arthritis.

Strengths of the Study

This review has several notable strengths. It specifically targeted a clinically challenging and increasingly recognized patient population, patients with rheumatoid arthritis who experience persistent pain despite well-controlled inflammatory activity, rather than the broader and more heterogeneous RA population. By focusing on this subgroup, the review addresses a clinically relevant gap in pain management that is not fully captured by studies primarily centered on inflammatory outcomes.

In addition, the review included randomized controlled trials, post hoc analyses of clinical trials, and large real-world observational cohorts, enabling a comprehensive assessment of the available evidence across diverse clinical contexts. Importantly, both pharmacological and non-pharmacological approaches were examined, encompassing not only structured exercise and physical therapy-oriented interventions but also observational evidence on lifestyle-related factors, such as dietary intake, that may influence pain outcomes independently of inflammatory control.

Furthermore, strict inclusion criteria were applied, and only studies with explicit definitions of low inflammatory activity and pain-related outcomes were included. This approach enhanced the clinical relevance of the findings and reduced interpretive ambiguity, while allowing consideration of multifactorial contributors to persistent pain beyond inflammation alone.

Limitations

Several limitations should be considered when interpreting the results of this review. First, the number of included studies was limited, and substantial heterogeneity existed in study design, sample size, intervention type, and outcome measurement. Definitions of low inflammatory activity and persistent pain varied across studies, limiting direct comparability. Second, evidence for non-pharmacological interventions was derived primarily from small, randomized pilot studies and observational follow-up studies, which may limit generalizability. Third, many pharmacological findings were based on post hoc analyses of randomized controlled trials that were not originally designed to evaluate pain outcomes in patients with controlled inflammation. As a result, the potential for selection and reporting bias cannot be excluded. Finally, comorbid conditions such as fibromyalgia, central sensitization, and psychological factors were not consistently assessed, limiting the ability to stratify patients according to pain phenotype.

## Conclusions

Persistent pain remains a significant clinical problem in patients with rheumatoid arthritis, even after successful control of inflammatory disease activity. The findings of this systematic review suggest that further escalation of pharmacological therapies targeting inflammation alone is often insufficient to resolve pain in this population. While specific agents, such as interleukin-6 receptor inhibitors, may offer modest benefits in selected patients, non-pharmacological interventions, particularly exercise-based and physical therapy-oriented approaches, demonstrate more consistent pain improvement independent of inflammatory control. Observational evidence also indicates that lifestyle-related factors, including dietary intake, may be associated with pain outcomes independently of inflammatory activity, further underscoring the multifactorial nature of persistent pain. Together, these results support a shift toward a more comprehensive, mechanism-informed approach to pain management in rheumatoid arthritis, integrating pharmacological treatment with non-pharmacological and lifestyle-oriented strategies tailored to individual pain profiles.
